# Notes on the genus *Gastrochilus* (Orchidaceae) in Myanmar

**DOI:** 10.3897/phytokeys.138.38781

**Published:** 2020-01-10

**Authors:** Qiang Liu, Shi-Shun Zhou, Ren Li, Yun-Hong Tan, Myint Zyaw, Xiao-Ke Xing, Jiang-Yun Gao

**Affiliations:** 1 Yunnan Forestry Technological College, Jindian Road, Panlong District, Kunming, Yunnan 650000, China Southeast Asia Biodiversity Research Institute, Chinese Academy of Sciences Nay Pyi Taw Myanmar; 2 Southeast Asia Biodiversity Research Institute, Chinese Academy of Sciences, Yezin, Nay Pyi Taw 05282, Myanmar Yunnan Forestry Technological College Yunnan China; 3 HponkanRazi Wildlife Sanctuary Offices, Putao, Myanmar HponkanRazi Wildlife Sanctuary Offices Putao Myanmar; 4 Institute of Medicinal Plant Development, Chinese Academy of Medical Sciences and Peking Union Medical College, China Institute of Medicinal Plant Development, Chinese Academy of Medical Sciences and Peking Union Medical College Beijing China; 5 Laboratory of Ecology and Evolutionary Biology, Yunnan University, Kunming, 650091, China Yunnan University Kunming China

**Keywords:** Orchidaceae, *
Gastrochilus
*, taxonomy, Myanmar

## Abstract

Myanmar is known for its high species richness of genus *Gastrochilus*; however, most of them lack proper information for taxonomic revision. During four years of field investigation in Myanmar, two new distributional records were encountered, namely, *G. arunachalensis* and *G. corymbosus* and one species, i.e. *G. pechei* was rediscovered after its original description. The three species were not easy to interpret from the available original descriptions and types due to severely shrunk or poorly preserved specimens. Therefore, we hereby present more detailed illustrations and updated descriptions for these species, based on freshly collected materials.

## Introduction

The genus *Gastrochilus* D. Don was established in 1825 (Epidendroideae; Vandeae; Aeridinae) and is characterised by saccate hypochile of the lip, a distinct epichile on the front of the saccate hypochile, two porate and globose pollinia borne on a slender stipe and a short axillary inflorescence, often with brightly coloured flowers (Christenson 1985; Seidenfaden 1988; Tsi 1996).

This genus includes around 62 species distributed from Andamans, Sri Lanka, India and the Himalayas eastwards to southern China, Indochina and southern Japan and southwards to the Philippines and Indonesia, of which many species are narrow endemics and there is a major centre of diversity in the South-East Asian archipelago (Chen et al. 2009; Kumar et al. 2014; Govaerts et al. 2016; Liu et al. 2016, 2019; Raskoti 2016; Averyanov et al. 2018; Liu and Gao 2018). According to the latest references, there are 9 species of *Gastrochilus* distributed in Myanmar (Kress et al. 2003; Kurzweil and Lwin 2014), but these are only known from very few, sometimes single, specimens or over-simplified descriptions.

During our field investigations in Myanmar since 2015, a total of eight species of *Gastrochilus* were discovered, of which two species were new records for Myanmar and *G. pechei* (Rchb.f.) Kuntze was collected more than 125 years after its first description in 1891 (Kuntze 1891). These three species are illustrated or recorded only from the type specimens and some of the key morphological characters were hard to interpret due to severely shrunk or poorly preserved specimens. Therefore, we hereby present more detailed colour illustrations and updated descriptions of these species based on fresh materials.

## Materials and method

Morphological observations of these species were based on living plants and 2–3 fertile specimens (kept in the herbaria of HITBC) or illustrations in original published papers (Das and Chanda 1988; Rao 1992). All morphological measurements were done by using a vernier calliper.

## Taxonomic treatment

### Gastrochilus arunachalensis

Taxon classificationPlantaeAsparagalesOrchidaceae

Nageswara Rao, (1992: 723)

795AA3DA-A5C7-5522-A7DF-5B43DCF792E9

Figure 1

#### Type.

INDIA. West Kameng Distinct, tropical rain forest, about 150 m a.s.l., epiphytic on tree trunks. A. N. Rao *24220* (holotype: Orchid Herbarium, Tipi!)

#### Description.

Epiphytic herbs. Stem, erect, 4.0 cm long and 1.0 cm in diameter, with 3–4 leaves. Leaves nearly basal, distichous, oblong, 8.0–15.0 × 1.7–2.3 cm, slightly fleshy or leathery, apex obtuse and unequally 2-lobed. 1–4 inflorescences from base of stem, sub-umbellate, often 8–10-flowered; peduncle straight, 1–2 cm, stout, with 2 cupular sheaths. Flower yellow or yellow green, with dark brown or purplish spots. Sepals similar, oblanceolate, 6.8–7.0 × 3.2–3.5 mm, base contracted, apex obtuse. Petals oblanceolate, 6.2–6.5 × 2.3–2.5 mm, apex obtuse. Lip with an epichile and a saccate hypochile; epichile triangular, 2.5–3.0 × 5.4–6.0 mm, fleshy, adaxially glabrous, with a central cushion, margin irregularly fimbriate or erose, apex rounded; hypochile cupular, ca. 6 mm tall, ca. 4 mm in diameter, white tinged with pale yellow at bottom, outside with 3 ridges. Column ca. 4 mm, stout; rostellum deeply 2–lobed; pollinia 2, ca. 1.0 mm in diam.; stipe elongate, ca. 2.0 mm; anther cap nearly subglobose, apex narrowed into a beak. Fruit cylindrical, ridged, 5–6 cm in length, 1.2–1.4 cm in diameter.

**Figure 1.**
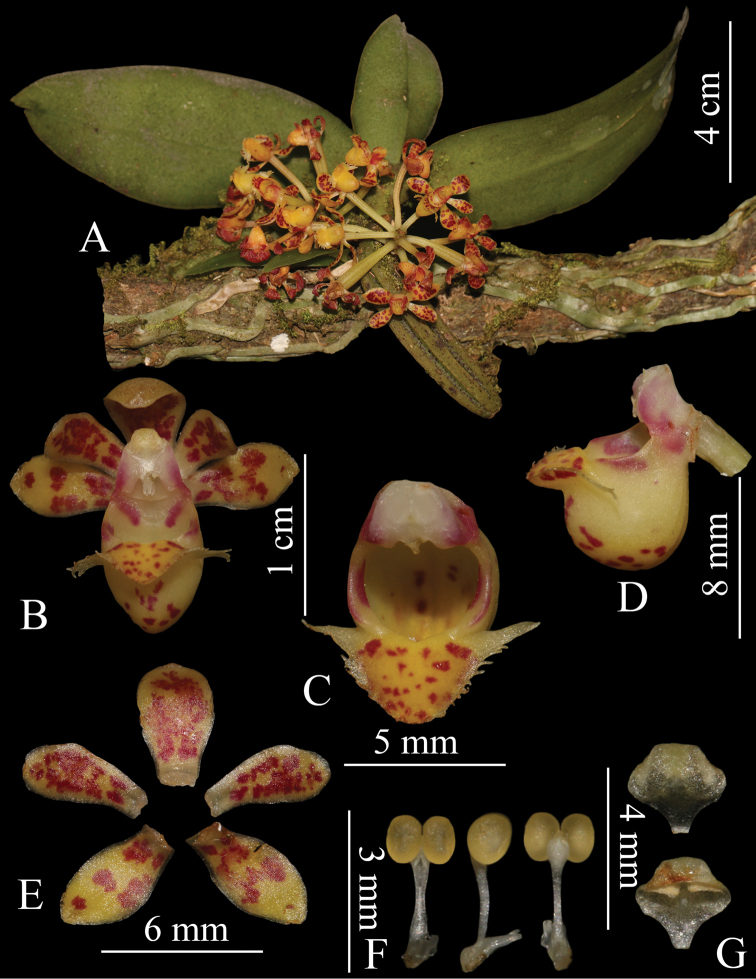
*Gastrochilus arunachalensis***A** habitat **B** front view of flower **C** front view of labellum and column **D** lateral view of labellum and column **E** sepals and petals; **F.** pollinarium **G** anther cap.

#### Phenology.

Flowering occurs in October and November.

#### Distribution and habitat.

*Gastrochilus arunachalensis* is previously known only from India and this is the first record from Myanmar (Putao county, Kachin state). It is epiphytic on the trunk of riparian trees in tropical rainforest in Myanmar or mixed deciduous tropical forest in India.

#### Additional specimens examined.

MYANMAR. Kachin State. Putao County, Wasadam village, tropical forest, 750 m a.s.l., epiphyte on the trunk of riparian trees, 14 Sep 2016, *Qiang Liu 408* (HITBC!). Kachin State. Putao County, Malirun village, tropical forest, 600 m a.s.l., epiphyte on the trunk of riparian trees, 29 Nov 2017, *Yun-Hong Tan M2965* (HITBC!).

#### Note.

*Gastrochilus arunachalensis* is an interesting species that was only known from the type specimen until recently, the key morphological characters of flowers being hard to interpret (Rao 1992). Now that fresh material has been collected in north Myanmar, a detailed description of the species, including the leaf and flower colour, leaf apex shape and features of pollinia, anther cap and rostellum, are provided here. Morphologically, it is closely related to *G. pechei* in having the sub-triangular epichile of the lip and hypochile of the lip laterally not compressed. However, it can be easily distinguished from the latter by the small stature of the plant (leaves less than 15 cm), green or yellow green flowers, oblanceolate sepals and petals, epichile triangular without central cushion (Kuntze 1891; Rao 1992).

### Gastrochilus corymbosus

Taxon classificationPlantaeAsparagalesOrchidaceae

A.P. Das & Chanda (1988: 401)

0D30E0A8-8B7B-5798-9BBA-9AC0F91ED2E2

Figure 2

#### Type.

INDIA. Jalapahar, Darjeeling (West Bengal), about 2200 m a.s.l., epiphytic on tree trunks. 29 Oct 1982, A.P. Das 823 (holotype: CAL!)

#### Description.

Epiphytic pendulous herb. Stem often branched, pendulous and usually 8.0–15.0 cm long with 0.4–0.5 cm internodes. Leaves distichous, blade oblong or falcate-lanceolate, 2.0–4.0 × 0.4–0.9 cm, apex acute and unequally 2-lobed. Inflorescence corymb, 4–6-flowered; peduncle 1.2–1.3 cm, upper part broader, lower part with 2 cupular sheaths with purple-red spots; floral bracts ovate-triangular, ca. 3.0 , membranous; pedicle and ovary yellow-green with purple-red spots, 1.0–1.2 cm long. Flowers yellowish or yellow, with purple blotches; epichile of lip white with sparse purple spots. Dorsal sepal oblong-elliptic, concave, 5.0–6.5 × 3.6–4.5 mm, apex obtuse; lateral sepal similar to dorsal sepal, 6.2–6.5× 3.2–4.0 mm, apex obtuse; petals sub-obovate, 5.5–6.5 × 3.5–4.2 mm, apex rounded. Lip with distinct partition between wide epichile and a saccate hypochile; epichile reniform, 4.0–4.9 × 8.0–9.0 mm, adaxially glabrous, with a slightly diamond-shaped central cushion covered with small brown spots and 2 conic calli near base, margin entire or slightly denticulate, emarginate at apex; hypochile cupular, laterally compressed, 7.2–7.8 mm tall, 5.8–6.2 mm in diameter, apex rounded. Column stout densely with purple spots, ca. 2 mm; anther cap galeate with recurved acuminate apex, 2.0 × 2.2 mm; pollinia 2, ca. 1.0 mm in diam.; stipe elongate, ca. 2.0 mm; rostellum bilobed with acuminate apex. Capsules cylindrical with 3 ridges, ca. 2.0 × 1.2 cm.

**Figure 2.**
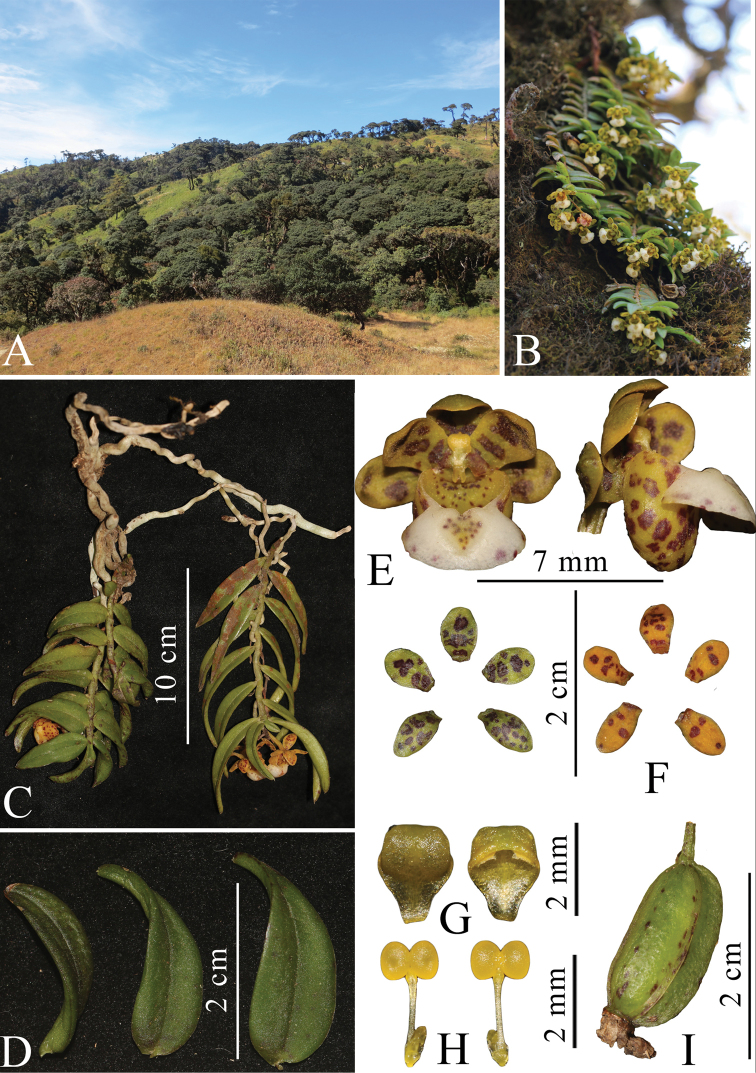
*Gastrochilus corymbosus***A, B** habitat **C** plant **D** leaf **E** front and lateral view of flower **F** sepals and petals **G** anther cap **H** pollinarium **I** fruit.

#### Phenology.

Flowering occurs in April to October.

#### Distribution and habitat.

*Gastrochilus corymbosus* is previously only known from the India and this is the first time that it has been recorded in the Natma Taung (Mt.Victoria) National Park, Chin State, Myanmar. It was found growing as an epiphyte on the tree trunks in a *Rhododendron* forest, which is dominated by Rhododendron protistum var. giganteum (Forrest) D.F. Chamberlain.

#### Additional specimens examined.

MYANMAR. Chin State. Natma Taung (Mt. Victoria) National Park, 2750 m a.s.l., epiphyte on the trunk of Alpine Rhododendron forests, 9 Jan 2017, *Qiang Liu 414* (HITBC!). Natma Taung (Mt.Victoria) National Park, 2900 m a.s.l., epiphyte on the trunk of Alpine Rhododendron forests, 30 Apr 2017, *Yun-Hong Tan M1271* (HITBC!).

#### Note.

Only a single specimen and illustration of this species previously existed, on which the original description was based (Das and Chanda 1988). Foliar and floral characteristics were not described in sufficient detail in the original description, due to the extremely poor state of the preserved specimen and it was not possible to conduct a detailed study on this species. Now that fresh material has become available, a detailed description of the species has been provided here. On the basis of the long and pendulous stem, *G. corymbosus* can be placed in section Microphyllae (Tsi 1996). This species exhibits great variation between populations in leaf shape from falcate-lanceolate to oblong (Figure 2 C). Morphologically, it shows a close affinity to *G. distichus* (Lindley) Kuntze, but differs from the latter by having much shorter (less than 15 cm) and stout stem, apex of leaf unequally 2-lobed without awns, inflorescences corymb with 4–6-flowers, epichile of lip with a slightly diamond shaped central cushion, margin entire or slightly denticulate, emarginate at apex (Das and Chanda 1988; Chen et al. 2009).

### Gastrochilus pechei

Taxon classificationPlantaeAsparagalesOrchidaceae

(Reichenbach f.) Kuntze (1891: 661)

BDDBE059-794C-56D1-B8DC-09F6F985599B

Figure 3

Saccolabium pechei Reichenbach f. (1889: 447) (Basionym)

#### Type.

MYANMAR. s. coll., s. n. (Holotype: K!)

#### Description.

Epiphytic herbs. Stem 1.0–1.5 cm, stout, with 4–5 leaves. Leaves nearly basal, distichous, oblong, 15–20 × 3.5–4.5 cm, slightly fleshy or leathery, apex obtuse and unequally 2-lobed. Inflorescences 1–4, from base of stem, sub-umbellate, often 4–6-flowered; peduncle straight, 1.5–2.5 cm, stout, with 2 cupular sheaths. Flower with yellow sepals and petals and white labellum, all dense with purplish spots. Sepals similar, spatulate, 11.8–12.5 × 4.2–5.4 mm, base contracted, apex obtuse. Petals spatulate, 11.5–11.7 × 4.0–4.2 mm, apex obtuse. Lip with an epichile and a saccate hypochile; epichile subtriangular, 5.0–5.2 × 15.1–15.4 mm, fleshy, adaxially glabrous, with a central cushion with a yellow blotch, margin irregularly erose, apex acute; hypochile subglobose, ca. 8 mm tall, ca. 8.4 mm in diameter, white tinged with yellow at bottom, outside with 5 ridges. Column ca. 3 mm, stout; rostellum deeply 2–lobed; pollinia 2, ca. 1.2 mm in diam.; stipe elongate, ca. 1.5 mm; anther cap nearly subglobose, apex narrowed into a beak.

**Figure 3.**
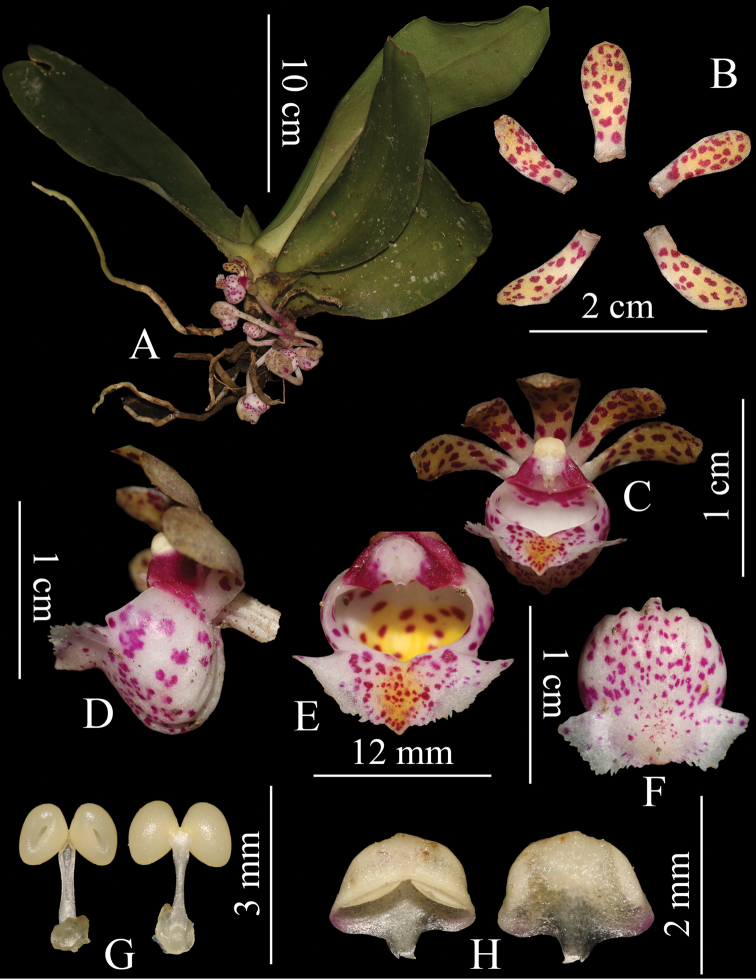
*Gastrochilus pechei***A** plant **B** sepals and petals **C** front view of flower **D** lateral view of flower **E** adaxial labellum **F** abaxial labellum **G** pollinarium **H** anther cap.

#### Phenology.

Flowering occurs in August and September.

#### Distribution and habitat.

*Gastrochilus pechei* was originally recorded from Myanmar without detailed information about locality. Here in the manuscript, we have confirmed the occurrence of this species in north Myanmar in the Naungmeng town, Putao county, Kachin state. It was growing epiphytically on the trunk of riparian trees in tropical rainforest which is dominated by *Dipterocarpus obtusifolius* Teijsm. ex Miq. (Dipterocarpaceae).

#### Additional specimens examined.

MYANMAR. Kachin State. Putao County, Naungmeng town, tropical forest, 700–800 m a.s.l., epiphyte on the trunk of riparian trees, 8 August 2017, *Qiang Liu 470* (HITBC!).

#### Note.

*Gastrochilus pechei* was only known from the type specimen until recently and, because this specimen was poorly preserved and severely shrunk, the key morphological characters of this species, such as flower colour and shape, were hard to interpret. As far as we can tell, there had been no subsequent collections of this species since 1889. Now that fresh material has become available, a detailed description of the species, including the plant and flower characters and information of distributed location, habitat and altitude, can be provided here. Morphologically, it shows a close affinity to *G. obliquus*, *G. somai* and *G. arunachalensis*. However, it differs from *G. obliquus* by having the slightly irregularly erose margin on the epichile, apex of epichile acute and subglobose hypochile (significantly lacerate or erose on epichile margin, apex of epichile obtuse and subglobose-cucullate and laterally compressed hypochile in *G. obliquus*) (Chen et al. 2009); from *G. somai*, it can be differentiated on being a large plant (leaves 15–20 × 3.5–4.5 cm), yellow sepals and petals and white labellum, all densely covered with purplish spots (smaller plant (3.5–4.2 × 1.2–1.7 cm), yellow-green sepals and petals without purplish spots and white labellum with yellow blotch on the centre of the epichile in *G. somai*) (Jin et al. 2010); from *G. arunachalensis*, by having spathulate sepals and petals, sub-triangular epichile with central cushion and subglobose hypochile (oblanceolate sepals and petals, triangular epichile without central cushion and cupular hypochile in *G. arunachalensis*) (Rao 1992).

#### Discussion.

The orchid flora of Myanmar is highly diverse but poorly known due to very few comprehensive studies. According to our current knowledge, about 800 orchid species are distributed in Myanmar (Kurzweil and Lwin 2014), but this is probably an underestimate. Many new distribution records and new species have been published in the last few years (Aung et al. 2017; Liu et al. 2017, 2018; Yang et al. 2017; Aung and Jin 2018, Zhou et al. 2018).

Although the genus of *Gastrochius* is small, it is easy to be confused with other taxa of Aeridinae when it is without flowers and is also difficult to be identified within species even during the flowering period. So, many species may be misidentified as other taxa. Meanwhile, Myanmar lies in southeast Asia, with the northern part bordering with southwest China and India and the southern part connecting with Thailand, all of these regions being rich in species of *Gastrochilus*. Therefore, we believe that more and more species of *Gastrochilu*s will be found when undertaking further field investigations and systematic studies.

## Key to the species of *Gastrochilus* D. Don in Myanmar

**Table:** 

1	Hypochile strongly dorsiventrally compressed from middle to tip, subtruncate and concave at tip	***G. platycalcaratus***
–	Hypochile subglobose or cupular, not dorsiventrally compressed	**2**
2	Hypochile subglobose and laterally compressed	***G. obliquus***
–	Hypochile subglobose or cupular, not compressed	**3**
3	Stem erect	**4**
–	Stem pendulous	**5**
4	Sepals and petals oblanceolate, epichile triangular without central cushion, epichile margin irregularly fimbriate or erose	***G. arunachalensis***
–	Sepals and petals spatulate, epichile subtriangular with central cushion, epichile margin lacerate or erose	***G. pechei***
5	Epichile densely haired adaxially and with a cavity at base	**6**
–	Epichile glabrous without cavity at base	**7**
6	Flowers size 2–3 cm in diam., hypochile subconic or subglobose	***G. bellinus***
–	Flowers size 1–1.8 cm in diam., hypochile galeate	***G. calceolaris***
7	Stem stout and leaf over 5 cm in length	**8**
–	Stem slender and leaf less 3 cm in length	**9**
8	Flower large (1.8–2.0 cm in diam.) and epichile margin entire	***G. acutifolius***
–	Flower small (0.6–0.8 cm in diam.) and epichile margin erose or irregular toothed	***G. intermedius***
9	Epichile sub-elliptic, without central cushion and conic calli	***G. pseododistichus***
–	Epichile sub-orbicular, with a central cushion, base with 2 conic calli	**10**
10	Short (less than 15 cm) and stout stem, leaf apex unequally 2-lobed without awns, inflorescences corymb with 4–6-flowered	***G. corymbosus***
–	Long (more than 30 cm) and slender stem, leaf apex with 2 or 3 awns, inflorescences subumbellate with 2–4-flowered	***G. distichus***

## Supplementary Material

XML Treatment for Gastrochilus arunachalensis

XML Treatment for Gastrochilus corymbosus

XML Treatment for Gastrochilus pechei
